# Anatomy of moist heatwaves in India during the summer monsoon season

**DOI:** 10.1007/s00382-025-08023-w

**Published:** 2026-02-16

**Authors:** Akshay Deoras, Andrew G. Turner, S. Lekshmi, Cathryn E. Birch, Ambrogio Volonté, Arathy Menon, Reinhard K. H. Schiemann, Laura J. Wilcox

**Affiliations:** 1 National Centre for Atmospheric Science, University of Reading, Reading, RG6 6EG UK; 2 Department of Meteorology, University of Reading, Reading, RG6 6EG UK; 3 Indian Institute of Tropical Meteorology, Pune, 411008 India; 4 School of Earth and Environment, University of Leeds, Leeds, LS2 9JT UK; 5 Met Office, FitzRoy Road, Exeter, EX1 3PB UK

**Keywords:** Moist heatwaves, Wet-bulb temperature, Indian summer monsoon, Active-break phases, BSISO

## Abstract

Moist heat impairs the human body’s ability to cool through sweat-based evaporative cooling, posing a serious health risk. In India, this risk is especially acute, since the Indian summer monsoon (ISM) brings abundant moisture, and socio–economic conditions significantly increase the exposure and vulnerability to moist heat. However, there is a limited understanding of the characteristics and large-scale drivers of moist heatwaves during the ISM. This study uses the ERA5 reanalysis to analyse moist heatwaves and their relationship with active and break periods of the ISM during 1940–2023. An empirical orthogonal function analysis of daily maximum wet-bulb temperature (T_w_) anomalies reveals that the first two principal components (PCs) explain key patterns of variability of moist heatwaves, with PC1 controlling their occurrence and PC2 controlling their spatial extent. Whilst breaks in the monsoon favour moist heatwaves in eastern and peninsular India, active rainfall events, corresponding to phases 5–7 of the Boreal Summer Intraseasonal Oscillation, favour moist heatwaves in northern and northwestern India. Specific humidity plays a larger role than dry-bulb temperature in controlling T_w_ variability in India. The results of this study reveal important characteristics of moist heatwaves during the ISM and offer potential for developing forecasting tools, which could ultimately benefit stakeholders in India.

**Supplementary Information:**

The online version contains supplementary material available at 10.1007/s00382-025-08023-w.

## Introduction

Moist heat, which encompasses the combined drivers of temperature and humidity, poses a severe threat to human health and wellbeing. The human body primarily cools itself through the evaporation of sweat, a process that becomes less effective as humidity rises (e.g., Alber-Wallerström and Holmér 1985). This is because high humidity impairs evaporation, limiting the human body’s ability to dissipate metabolic heat and regulate core temperature. In such circumstances, the physiological strain on the human body intensifies, exacerbating the risks of cardiovascular and respiratory illnesses (e.g., Donaldson et al. 2003; Liu et al. 2022). A thermoregulatory failure can lead to hyperthermia, heat exhaustion, and fatal heatstroke in extreme cases (e.g., Sherwood and Huber 2010; Hanna and Tait 2015; Buzan and Huber 2020). These health impacts can cascade into broader socio-economic consequences, especially in densely populated and economically vulnerable countries such as India. The Indian summer monsoon (hereafter: the monsoon) is the main source of moisture between June and September, and it plays a key role in driving extreme moist heat across the country (Raymond et al. 2020). Extreme moist heat stress is rapidly intensifying globally (Raymond et al. 2020), with India experiencing a rising frequency and intensity of moist heat days (Mishra et al. 2020; Rogers et al. 2021). These trends are expected to continue under future warming scenarios (Im et al. 2017; Matthews 2018; Murari and Ghosh 2018; Saeed et al. 2021), posing a growing threat to over a billion people in the country, particularly agricultural workers, labourers and the elderly. It is therefore crucial to understand the characteristics of moist heat in India during the monsoon and identify its large-scale drivers.

Many indices have been developed to measure moist heat stress, including, for example, the Heat Index, Universal Thermal Climate Index, and Wet-Bulb Globe Temperature (e.g., Minard 1961; Steadman 1979; Jendritzky et al. 2012). However, it is unclear which metric is best suited for health impacts. The wet-bulb temperature (T_w_) continues to be a commonly used thermodynamic variable for assessing moist heat stress, since it can be measured directly (i.e., without relying on approximations) and it directly reflects how effectively the human body can cool itself through sweating (Sherwood and Huber 2010). Among these indices, humidity has the strongest control on T_w_ (Sherwood 2018), and T_w_ contrasts more strongly with dry heat (i.e., dry-bulb temperature) than others. For a given combination of humidity and ambient temperature, T_w_ represents the lowest temperature that can be achieved through evaporative cooling. A T_w_ threshold of 35 °C has traditionally been considered as the upper physiological limit for human survivability under sustained exposure, beyond which the human body can no longer maintain a stable core temperature (e.g., Sherwood and Huber 2010; Pal and Eltahir 2016; Schär 2016). Prolonged exposure to such conditions for as little as six hours can be fatal (Sherwood and Huber 2010). However, recent advances in biophysical modelling have revealed that the 35 °C T_w_ threshold may overestimate human heat tolerance, especially across different age groups, exposure durations, and environmental conditions. Vanos et al. (2023) demonstrated that realistic survivability thresholds vary substantially, with updated limits ranging from 25.8 to 34.1 °C for younger adults and 21.9–33.7 °C for older adults. Notably, older female adults exhibit the lowest tolerance, with survivability thresholds in dry conditions estimated to be up to 7 °C to 13 °C below the conventional 35 °C benchmark. Since T_w_ does not account for variations in solar exposure, it overlooks the influence of direct sunlight on heat stress. As a result, T_w_ may slightly overestimate heat stress under cloudy conditions or at night, while underestimating it during periods of intense solar radiation (Im et al. 2017). Besides, it does not account for wind speed as in the Wet-Bulb Globe Temperature and Universal Thermal Climate Index.

Prior to the monsoon onset, India experiences high surface temperatures and dry heatwaves, which have been extensively studied. Ratnam et al. (2016) performed an empirical orthogonal function (EOF) analysis of maximum surface temperature anomalies over India during March–June. They identified two types of dry heatwaves over the country. The first type occurs over north-central India, which is associated with an atmospheric blocking pattern over the North Atlantic. The second type occurs over the east coast of India, which is attributed to the anomalous Matsuno-Gill response to the anomalous cooling in the Pacific Ocean. The spatial patterns of these first two EOF modes were also obtained by Lekshmi and Chattopadhyay (2022), who performed an EOF analysis of surface temperature anomalies in India during April–May. Lekshmi and Chattopadhyay (2022) and Lekshmi et al. (2024) found that the second EOF mode represents a regional circulation pattern that supports moisture transport from the Bay of Bengal to the east coast of India, driving moist heat extremes there.

Raymond et al. (2020), Rogers et al. (2021), and Ivanovich et al. (2024) have shown that moist heat in India and other parts of South Asia often occurs following the monsoon onset in June. Ivanovich et al. (2024) found that most extreme T_w_ events in India occur on rainy days. However, the intensification of T_w_ in a region depends on the background humidity. In climatologically drier regions, positive T_w_ anomalies tend to occur when precipitation is enhanced (i.e., during wet spells or an early onset of the monsoon), whereas in climatologically humid regions, suppressed precipitation during dry spells or a delayed monsoon onset leads to an increase in positive T_w_ anomalies. In such cases, increased surface heating and reduced evaporative cooling contribute to an increase in T_w_. However, a limitation of Ivanovich et al. (2024) is that dry and wet spells were defined locally, which might not always reflect wet and dry spells of the monsoon over the core monsoon zone (a region in central India characterised by Rajeevan et al. 2010) that are tied to large-scale circulation anomalies (e.g., Pai et al. 2016). Besides, Ivanovich et al. (2024) did not explicitly identify moist heatwaves. Jackson et al. (2025), who examined the relationship between precipitation and moist heat in the tropics and subtropics, also obtained similar results. In moisture-limited environments (e.g., northwest India), moist heatwaves occur after enhanced precipitation, whereas in energy-limited environments (e.g., east India), they are associated with suppressed precipitation.

The subseasonal variability of rainfall is a defining characteristic of the monsoon (e.g., Krishnamurthy and Shukla 2000), and is strongly modulated by the Madden–Julian Oscillation (MJO) and the Boreal Summer Intraseasonal Oscillation (BSISO; e.g.,Kikuchi et al. 2012; Pai et al. 2016; Singh and Bhatla 2019). Whilst the eastward-propagating MJO dominates tropical subseasonal variability during boreal winter, the BSISO is the prevailing mode during boreal summer (e.g., Wang and Xie 1997). Ivanovich et al. (2022) used the all-season outgoing longwave radiation-based MJO index (OMI; Kiladis et al. 2014) to examine the influence of tropical intraseasonal oscillations (ISOs) on T_w_ in the Persian Gulf and South Asia. Unlike BSISO-specific indices (e.g., Kikuchi et al. 2012) that explicitly isolate the northward component of the ISO, the OMI captures both the MJO and BSISO modes. They found that extreme T_w_ in eastern and northwestern India is most likely to occur during phases 1–3 and 6 of the ISO, respectively. In phases 1–3 of the ISO, convection is mostly suppressed over eastern India, whereas it is enhanced over northwestern India in phase 6 (see their Fig. 4). They considered a T_w_ threshold of 28 °C, and found that its occurrence over eastern India is almost twice as likely during certain phases (e.g., phase 1) of the ISO than in others. They also found that anomalously high T_w_ in northwestern India closely aligns with positive specific humidity anomaly, which is associated with the convectively active phase of the ISO. In contrast, over the southeastern coast of India, high T_w_ is associated with convectively inactive phases of the ISO, suggesting the role of increased surface insolation and reduced evaporative cooling during monsoon breaks.

Our understanding of dry heat in India is much more comprehensive than our understanding of moist heat, which is also the case across many other regions such as East Asia (e.g., Ha et al. 2022), Southeast Asia (Lyu et al. 2024), southern China (Luo et al. 2022), and Africa (Birch et al. 2022). As a result, several key questions arise that are critical for advancing our knowledge of moist heat events and improving their predictability in India. Firstly, what are the spatio-temporal patterns of variability in moist heat across India? This question is important due to a vast demographic heterogeneity in India (Azhar et al. 2017), which could lead to significant regional disparities in exposure and vulnerability to moist heatwaves. Secondly, how are moist heatwaves influenced by active and break phases of the monsoon? This will help improve our understanding of the interplay between temperature and moisture during different phases of the monsoon. Lastly, in what ways does the BSISO modulate the occurrence of moist heatwaves in India? This is important for the predictability of moist heatwaves, since the predictive skill of the BSISO is about two to four weeks (Jie et al. 2017). We attempt to answer these questions in this study, for which we consider T_w_ as the measure of moist heat stress.

We present an outline of the data and methodology in Sect. 2, and explore the climatology of daily maximum and extreme T_w_ in Sect. 3. We then analyse characteristics of moist heatwaves in Sect. 4, and examine their relationship with active-break phases of the monsoon in Sect. 5. We investigate the role of the BSISO in modulating the occurrence of moist heatwaves in Sect. 6, and finally conclude in Sect. 7.

## Data and methodology

### ERA5 reanalysis

We use data from the European Centre for Medium-Range Weather Forecasts ERA5 reanalysis (Hersbach et al. 2020) to compute T_w_, identify moist heatwaves, and analyse precipitation and moisture flux in the lower troposphere. The ERA5 data are available globally from 1940 on a 0.25° × 0.25° grid and at an hourly temporal resolution. We consider data for the period June–September 1940–2023. Mahto and Mishra (2019) compared ERA5 precipitation with the Climate Forecast System Reanalysis, Modern Era Retrospective Analysis for Research and Applications version 2, ERA-Interim, and Japanese 55-year reanalysis datasets. They found that ERA5 outperformed other reanalyses for monsoon precipitation, which makes it suitable for our analysis. All variables considered in this study are instantaneous, except for precipitation that is accumulated hourly. Anomalies are calculated against a daily climatology during June–September 1940–2023.

### IMD gridded precipitation data

We use the fourth version of the daily gridded precipitation dataset from the India Meteorological Department (IMD; Pai et al. 2014), which is available from 1901 at a spatial resolution of 0.25° × 0.25°. It uses data from rain gauges across India, whose number has varied in time from about 1450 in 1901 to about 3950 during the period 1991–1994. However, daily data from about 2600 rain gauge stations were available over the last century (Pai et al. 2014). A simple inverse distance-weighted interpolation method (Shepard 1968) is used to regrid the gauge data. Whilst the spatial distribution and seasonal cycle of monsoon rainfall are well captured, the low spatial density of rain gauges in hilly regions of northern and northeastern India makes the dataset less reliable there (Pai et al. 2014). Given that this dataset is based on rain gauges, it is available only for mainland India, so it cannot be used to analyse precipitation over the Arabian Sea and Bay of Bengal.

### BSISO indices

We consider the BSISO indices from the International Pacific Research Centre, whose methodology is described in Kikuchi and Wang (2010), Kikuchi et al. (2012), and Kikuchi (2020). They are derived by performing an extended EOF analysis of outgoing longwave radiation. The daily BSISO index contains the normalised values of the first two principal components (PCs), as well as the resulting phase and amplitude. The indices are available for the period 1979–2021.

### Calculation of T_w_

We consider the Davies-Jones method (Davies-Jones 2008) to calculate T_w_ at 2 m using data from the ERA5 reanalysis. It is based on an iterative process that uses the Bolton’s equivalent potential temperature formula (Bolton 1980) to determine T_w_ along a given pseudoadiabat and pressure level. The computation starts with an initial guess of T_w_ and then refines the value through iterations. Compared to other methods (e.g., Stull 2011), the Davies-Jones method is considered to be more efficient and reliable (Buzan and Huber 2020). Besides, it is more accurate at high temperatures and is widely used for research on T_w_ extremes (Sherwood and Huber 2010; Coffel et al. 2017, 2019; Raymond et al. 2020).

### Calculation of heat index

We calculate the adjusted Heat Index to test the sensitivity of some of our results to the choice of the metric used to measure moist heat stress. Heat Index combines air temperature and relative humidity to determine an apparent temperature that indicates how hot it actually feels. The Heat Index equation, originally developed by Rothfusz (1990), was derived using multiple regression analysis, incorporating temperature and relative humidity data from the initial version of Steadman’s apparent temperature model (Steadman 1971). The adjusted Heat Index modifies the standard Heat Index by factoring in additional environmental or situational elements that affect how heat is experienced. It is considered to be more accurate than the Heat Index in the tropics (Brimicombe et al. 2022).

### Identification of moist heatwaves

We follow Jackson et al. (2025) for identifying moist heatwaves at each grid point during June–September 1940–2023.^1^We first calculate the 95th percentile of daily maximum T_w_ at each grid point. The 95th percentile captures local seasonality and regional variations in T_w_ (Jackson et al. 2025). We define the occurrence of a moist heatwave at a grid point when the daily maximum T_w_ exceeds a minimum threshold of 24 °C and the 95th percentile threshold for at least three consecutive days. The minimum threshold of 24 °C represents a T_w_ that is low risk for most people (Lo et al. 2021; Vanos et al. 2023). Moist heatwaves occurring over the Arabian Sea, Bay of Bengal, and where the mean surface pressure over land is less than 850 hPa (i.e., high mountain regions) are disregarded.

### Identification of active and break phases of the monsoon

**Fig. 1:**
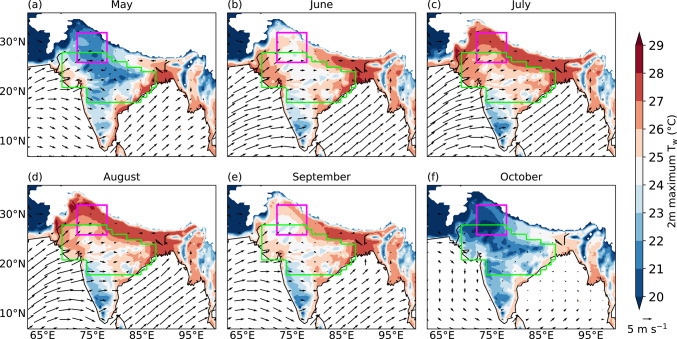
Climatology of daily maximum 2 m wet-bulb temperature (T_w_; °C) and 10 m winds (vectors; m s^−1^) during 1940–2023, calculated using the ERA5 reanalysis. Magenta box shows the north India domain (26°–32°N, 72°–78°E) and the core monsoon zone is highlighted in green. Grid points over the north Indian Ocean and where the mean surface pressure is less than or equal to 850 hPa are masked.

We use the IMD gridded precipitation dataset to identify active and break phases of the monsoon during June–September 1940–2023,^2^ for which we follow the method proposed by Rajeevan et al. (2010). An event is classified as an active event if the standardised precipitation anomaly over the core monsoon zone (shown in magenta in Fig. 1a) is greater than 1 for at least three consecutive days. Conversely, if it is less than −1 for at least three consecutive days, a break phase is identified. We identify 261 active events and 188 breaks in total, whose mean durations are 4.4 and 5.5 days, respectively. The frequency of active and break events is maximum during August and least in June (not shown). We identify the middle day of each break and active event, based on whether their duration is an even or odd number of days. For example, if a break or an active event occurs for five days, its middle day is defined as the third day from its onset. If the duration is four days, either the second or third day is chosen depending on precipitation over the core monsoon zone: for breaks, the day with less precipitation is selected, whereas for active events, the day with more precipitation is chosen as the middle day. There are 1045 break days and 1143 active days in total in our analysis period.

### Moisture flux transport

In order to diagnose moisture transport in the lower troposphere, we calculate the vertically integrated moisture flux transport (VIMF) between 1000 and 700 hPa as follows:

$$VIMF = \frac{1}{g}{\int }_{1000}^{700}({\boldsymbol{u}} q) dp$$

where ***u*** is the horizontal wind vector, *q* is specific humidity, and other symbols have their usual meanings. Anomalies of VIMF are calculated against a daily climatology for June–September 1940–2023.

### Significance testing

We use a Student’s *t* test (Student 1908) to assess if anomalies of T_w_ and precipitation are significantly different from zero. Our null hypothesis states that anomalies are not significantly different from zero. Thus, areas where anomalies are significantly different from zero at the 95% confidence level are stippled.

### Domains

Throughout this study, we focus on the following two domains in India: north India (26°–32°N, 72°–78°E; shown as a magenta box in Fig. 1) and the core monsoon zone (highlighted in green in Fig. 1). The rationale for selecting these domains is discussed in subsequent sections.

## Climatology of daily maximum and extreme T_w_

**Fig. 2:**
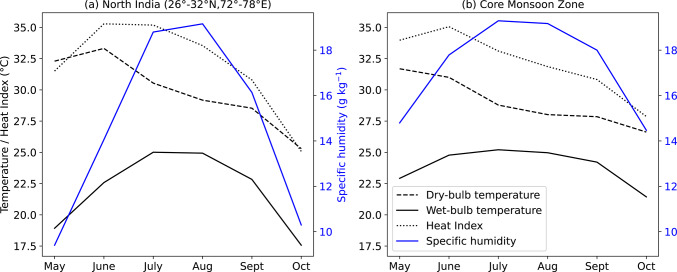
Climatology of daily mean dry-bulb temperature (dashed black; °C), wet-bulb temperature (solid black; °C), Heat Index (dotted black; °C) and specific humidity (solid blue; g kg^−1^) at 2 m calculated over **a** north India domain (26°–32°N, 72°–78°E) and **b** the core monsoon zone during 1940–2023.

In this section, we analyse the climatology of daily maximum T_w_ at each grid point in order to understand how T_w_ varies during the monsoon. During May, the maximum T_w_ reaches approximately 28 °C along the east coast, representing the highest across the country at that time (Fig. 1a). In northern and central India, T_w_ is less than 25 °C. The low magnitude of T_w_ is indicative of the dry weather conditions during the pre-monsoon period, which can be seen from the low magnitude of daily mean 2 m specific humidity during May (Fig. 2). As the monsoon progresses in June, T_w_ increases across most of India, with maximum T_w_ exceeding 29 °C in some parts of eastern India (Fig. 1b). The low-level moist monsoon winds, which arrive from the Arabian Sea and Bay of Bengal, supply moisture to the country, increasing 2 m specific humidity (Fig. 2). T_w_ increases further in July and August (Fig. 1c, d), reaching its highest values in the Indo-Gangetic Plains (IGP) and northwestern India. Over the core monsoon zone, the maximum T_w_ varies between 23 and 28 °C (Fig. 1c, d), whereas the mean T_w_ is around 25 °C (Fig. 2b). It begins to decline from September as the monsoon starts withdrawing from the country (Figs. 1e and 2). Whilst it continues to exceed 27 °C over eastern India (Fig. 1e), it falls below 27 °C over northern India. By October, low-level monsoon winds weaken and no longer supply moisture to most of the country, resulting in a further reduction in specific humidity (Fig. 2) and T_w_. In fact, maximum and mean T_w_ reduce to below 20 °C in northern India (Figs. 1f and 2a). Interestingly, T_w_ over the Deccan Plateau in southwestern India remains below 25 °C during May–October, indicating either low surface temperatures, limited surface humidity, or both. The linear relationship between specific humidity and T_w_ is evident from Fig. S1, which shows scatter plots of monthly mean 2 m dry-bulb temperature versus 2 m T_w_ (Fig. S1a, c) and 2 m specific humidity versus 2 m T_w_ (Fig. S1b, d) over the north India domain and the core monsoon zone for the period May–October 1940–2023. This differs from the non-linear relationship between 2 m dry-bulb temperature and 2 m Tw in the same period.

We also analyse the monthly mean of extreme T_w_ (i.e., the 95th percentile of daily maximum T_w_ at each grid point occurring within a given month across the whole time series). During July and August, extreme T_w_ in some parts of northern India and eastern Pakistan exceed 30 °C, whereas it is above 29 °C across the IGP and eastern India (Fig. S1b, c). Over most of the core monsoon zone, it is 26°–28 °C and less than 26 °C over peninsular India except the southeast coast. It continues to be around 30 °C along the IGP and east India during September (Fig. S2d). These findings align with those of Ivanovich et al. (2024), who found that the 95th percentile of daily mean T_w_ in northern India ranges from 28 °C to 31 °C and daily maximum T_w_ often exceeds this range. Since extreme T_w_ values of 29 °C and above are generally associated with severe heat stress (Vanos et al. 2023), they are expected to significantly increase the risk of heat-related illnesses, particularly among vulnerable populations in these regions.

In summary, increased surface humidity during the monsoon leads to elevated maximum T_w_ across India, with the highest T_w_ occurring during the peak monsoon months of July and August. Among all regions, the IGP and northern India consistently record the highest T_w_, often exceeding 29 °C.

## Characteristics of moist heat in India

We now examine characteristics of moist heat. We first identify the spatio-temporal patterns of variability in moist heat by performing an EOF analysis of daily maximum T_w_ anomaly. We then analyse how the occurrence of moist heatwaves and precipitation varies in relation to these EOF patterns.

### Spatio-temporal modes of variability of T_w_

**Fig. 3:**
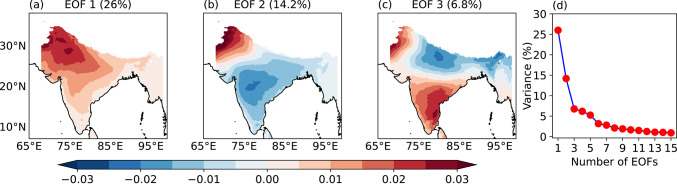
Spatial pattern of the first three EOFs of daily maximum 2 m wet-bulb temperature anomaly during June–September 1940–2023 (**a**–**c**) and variance explained (%) versus number of EOFs (**d**). Numbers in brackets show the percentage variance explained by each EOF mode. The EOF analysis is performed only over land, and grid points where the mean surface pressure is less than or equal to 850 hPa are masked.

We perform an EOF analysis of daily maximum T_w_ anomaly in India (7°–36°N, 68°–98°E). Each PC time series quantifies how strongly a specific spatial pattern appears on any given day. Thus, positive and negative PC values represent different phases of the same spatial pattern, which are often associated with different meteorological conditions. The daily maximum T_w_ anomaly at each grid point was detrended to remove long-term trends before computing EOFs. Figure 3a–c show the spatial pattern - of the first three EOF modes. The first EOF mode explains 26% variance (Fig. 3d) and features a monopole pattern. The second EOF mode explains 14.2% variance and features a dipolar pattern, with opposite signs of T_w_ anomaly between northwest India and the rest of the country. Thus, the spatial patterns of the first two EOF modes of T_w_ are similar to those of dry-bulb temperatures during the pre-monsoon period (Ratnam et al. 2016; Lekshmi and Chattopadhyay 2022), and therefore, we focus on these two modes in this study. We extract the first two normalised PCs and analyse their lead-lag correlation to understand if they are coupled. We find a very weak positive correlation at a lag of 5 days, suggesting that they are not coupled (Fig. S3). The third EOF mode explains only 6.8% variance and features a tripolar pattern (Fig. 3c); this EOF mode could be further examined in a future study. We perform an additional EOF analysis using daily mean T_w_ (not shown), since this metric is also valuable for assessing moist heat stress. The spatial patterns of the first two EOF modes derived from daily mean T_w_ closely resemble those obtained from the EOF analysis of daily maximum T_w_.

**Fig. 4:**
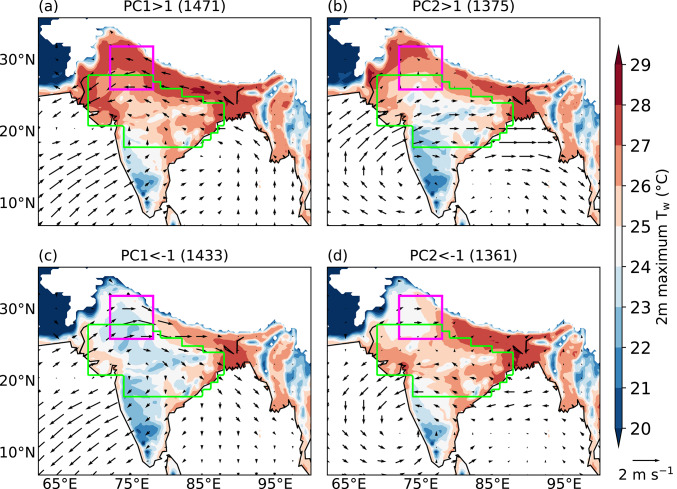
Composite daily maximum 2 m wet-bulb temperature (T_w_; °C) at each grid point and 10 m wind anomalies (vectors; m s^−1^) for various magnitudes of the first two principal components (PCs) during June–September 1940–2023. Numbers in brackets show the number of days in each category. The north India domain and core monsoon zone are highlighted in magenta and green, respectively. Grid points over the ocean and where the mean surface pressure is less than or equal to 850 hPa are masked.

Lekshmi et al. (2024) analysed composites of the circulation and surface temperature for days when the amplitude of PC1 and PC2 exceeded one standard deviation. This approach helps identify days that are associated with an increased risk of dry or moist heat stress, which could benefit stakeholders such as public health officials and other government authorities. Here, we adapt the same framework to diagnose moist heat and analyse maximum T_w_ and 10 m wind anomaly. For PC1 > 1, the maximum T_w_ across some parts of the IGP exceeds 28 °C, accompanied by anomalous easterly winds over the region (Fig. 4a). The anomalous wind pattern over the Arabian Sea and Bay of Bengal is favourable for the enhanced transport of moisture towards India. Over the core monsoon zone, the maximum T_w_ ranges from 23 to 29 °C, with lowest value in the southern part of the region. In contrast, on days when PC1 < − 1, the maximum T_w_ is generally lower across the core monsoon zone and northern India, though its magnitude is still around 28 °C over Bangladesh and the eastern IGP (Fig. 4c). The wind anomaly pattern indicates a weakened monsoon circulation, which could be limiting the transport of moisture towards India, especially to southern India and the IGP. The maximum T_w_ over northern India is higher on days when PC2 > 1 (Fig. 4b) compared to those when PC2 < − 1 (Fig. 4d). In contrast, the maximum T_w_ over the core monsoon zone and eastern India is higher when PC2 < –1, during which anomalous winds feature an easterly pattern from the Bay of Bengal. We also analyse maximum 2 m T_w_ for combinations of PC1 and PC2 featuring their amplitude between − 1 and 1 (not shown). For these PC combinations, the maximum T_w_ in the Indo-Gangetic plains is approximately 28 °C, whereas in the core monsoon zone, it varies between 23 and 28 °C. Besides, the 10 m wind anomalies are small in magnitude. The spatial pattern of moist heatwave occurrence is largely uniform across all combinations.

**Fig. 5:**
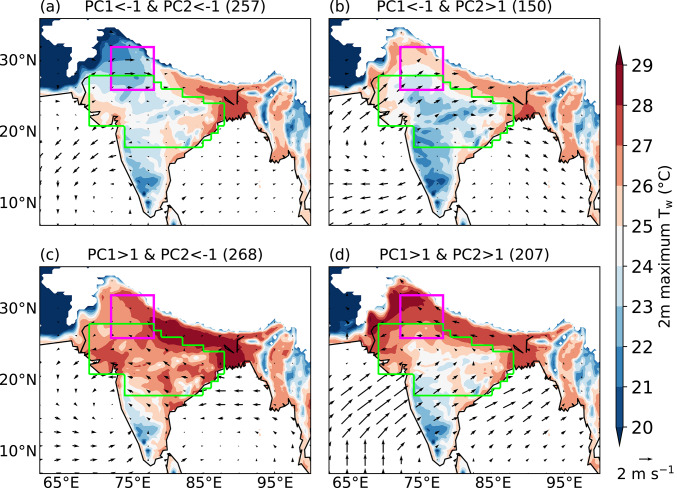
As in Fig. 4, but showing combinations of PC1 and PC2 of daily maximum T_w_. Vectors show 10 m wind anomalies (m s.^−1^).

We now analyse the maximum T_w_ by considering the combined behaviour of PC1 and PC2. On days when PC1 < − 1, regardless of the magnitude of PC2, the maximum T_w_ remains relatively low across most of India (Figs. 5a, b), except across the IGP and eastern India. Specifically, when PC1 < − 1 and PC2 < − 1 (Fig. 5a), the maximum T_w_ drops below 22 °C in northern India, indicating minimal risk of moist heat stress there. In contrast, when PC1 > 1 and PC2 < − 1 (Fig. 5c), the maximum T_w_ across most of the country is elevated, exceeding 28 °C over the IGP and eastern India, and reaching up to 28 °C across the core monsoon zone and northern India. On days when the normalised magnitude of both PC1 and PC2 exceeds one (Fig. 5d), maximum T_w_ decreases over the core monsoon zone while increasing over northern India and eastern Pakistan, often surpassing 28 °C. A box-and-whisker plot of the maximum T_w_ over northern India for various combinations of PC1 and PC2 is shown in Fig. S4. The spatial patterns are similar when daily maximum Heat Index is considered instead of daily maximum T_w_ (Fig. S5). The Heat Index across most of the country is elevated when PC1 > 1 and PC2 < − 1 (Fig. S5c) compared to the other three clusters. It is highest across northern India (exceeding 44 °C) when the normalised magnitude of both PC1 and PC2 exceeds one (Fig. S5d). This suggests that our results are not strongly dependent on the specific moist heat stress metric used. We therefore proceed with a focus on T_w_ in the subsequent section.

We also analyse the absolute and anomalous mean T_w_ for these four combinations. The mean T_w_ over the core monsoon zone is highest when PC1 > 1 and PC2 < − 1, ranging between 22 °C near the Deccan Plateau and 27 °C near the IGP (Fig. S6c). Over northern India, the mean T_w_ ranges from 24 to 27 °C when PC1 > 1 and PC2 > 1 (Fig. S6d). The mean T_w_ across the country is much lower for other PC combinations (Fig. S6a, b), agreeing with the results of maximum T_w_. We also investigate anomalies of mean dry-bulb temperature and mean specific humidity at 2 m to understand the relative contributions of temperature and moisture to the mean T_w_ pattern (Figs. S7, S8). Over northern India, when PC1 < − 1 and PC2 < − 1, the dry-bulb temperature anomaly exceeds 2 °C (Fig. S7a), while the specific humidity anomaly is less than − 4 g kg^−1^ (Fig. S8a). When PC1 > 1 and PC2 < − 1 (Figs. S7c, S8c), most of the core monsoon zone and northern India experience positive anomalies in both dry-bulb temperature (up to 1 K) and specific humidity (up to 2 g kg^−1^). In contrast, when both PC1 > 1 and PC2 > 1, the dry-bulb temperature anomaly becomes negative (Fig. S7d), while the specific humidity anomaly remains positive over both the core monsoon zone and northern India (Fig. S8d). In fact, the specific humidity anomaly exceeds 3 g kg^−1^ over northern and northwestern India (Fig. S8d). These results suggest that, in general, specific humidity plays a greater role than dry-bulb temperature in modulating the T_w_ pattern.

### Occurrence of moist heatwaves

**Fig. 6:**
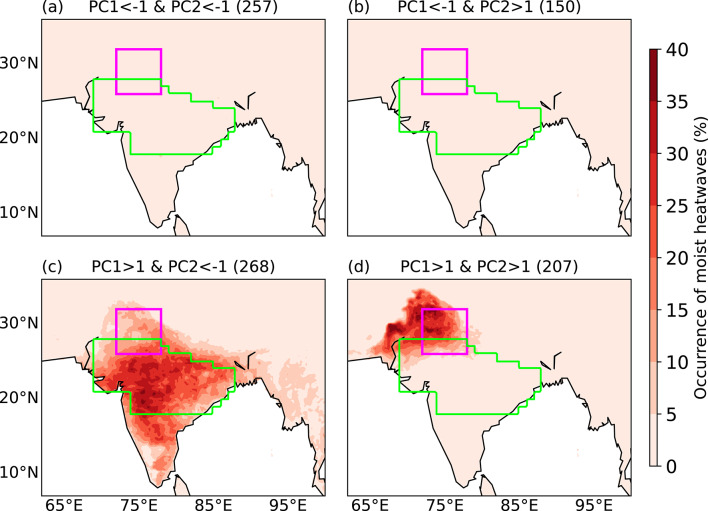
Percentage occurrence of moist heatwave days at each grid point for various combinations of principal components (PCs) during June–September 1940–2023. The occurrence of a moist heatwave at a grid point is defined when the daily maximum wet-bulb temperature exceeds a minimum threshold of 24 °C and the 95th percentile threshold for at least three consecutive days. The percentage occurrence at each grid point is calculated by normalising the total number of moist heatwave days by the total number of days in that PC combination (shown in brackets). The north India domain and core monsoon zone are highlighted in magenta and green, respectively.

Having analysed how the maximum and mean T_w_ in India vary in relation to the two PCs, we now examine how the occurrence of moist heatwaves is modulated by these PCs (Fig. 6). As discussed in Sect. 2.6, we define the occurrence of a moist heatwave at a grid point when the daily maximum Tw exceeds a minimum threshold of 24 °C and the 95th percentile threshold for at least three consecutive days. Since the number of days corresponding to each PC combination differs, we normalise the number of moist heatwave days at each grid point by the total number of days of occurrence of that PC cluster. Moist heatwaves do not occur in India when PC1 < –1, regardless of the sign of PC2 (Figs. 6a, b). In contrast, they are prevalent in different regions of India when PC1 > 1. When PC1 > 1 and PC2 < − 1, moist heatwaves occur across many regions of India, with the highest relative frequency of moist heatwave days reaching up to 40% over the core monsoon zone (Fig. 6c). However, when both PC1 > 1 and PC2 > 1, moist heatwaves are largely confined to northern India and adjoining parts of eastern Pakistan (Fig. 6d). These results suggest that whilst PC1 plays a critical role in the occurrence of moist heatwaves, PC2 modulates their spatial distribution. For the sake of simplicity in this work, we refer to the “PC1 < − 1 and PC2 < − 1” and “PC1 < − 1 and PC2 > 1” clusters as “low risk” clusters. The “PC1 > 1 and PC2 < − 1” and “PC1 > 1 and PC2 > 1” clusters are referred to as the “pan-India moist heat stress” and “northwest India moist–heat stress” clusters, respectively.

We also analyse the occurrence of moist heatwaves for combinations of PC1 and PC2 featuring their amplitude between –1 and 1 (not shown). The spatial pattern of moist heatwave occurrence is largely uniform across all combinations. There is moist heat stress on 4–5% of total days in the respective PC combinations in the northwest Indian region and a few coastal areas. Since the total number of days in the respective PC combinations is ~ 7000, there are ~ 300 days of moist heat stress. As discussed in Sect. 4.1, the first two EOF modes together explain ~ 40% of variance in the data (Fig. 3) and the associated moist heat stress events. Therefore, we focus our analysis on these dominant modes that contribute most strongly to moist heat stress. We acknowledge, however, that moist heat stress can also arise from other modes, which we do not investigate in this work.

### Precipitation and moisture transport

**Fig. 7:**
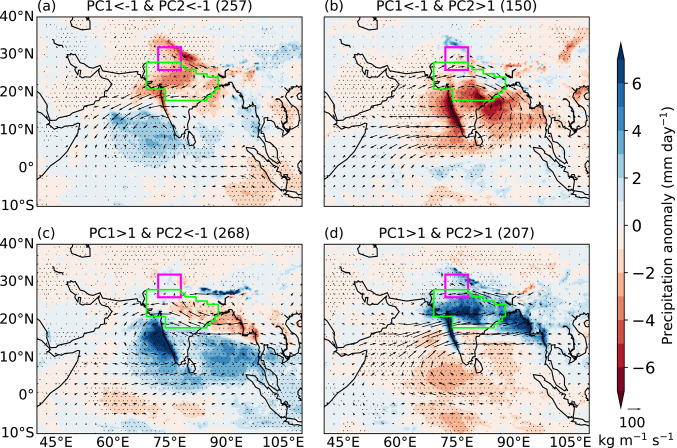
Anomalies of daily mean precipitation (shading; mm day^−1^) and 1000–700 hPa vertically integrated moisture flux (vectors; kg m^−1^ s^−1^) for various combinations of the first two principal components (PCs) during June–September 1940–2023. The anomalies are computed against a daily climatology during June–September 1940–2023. Stippling shows regions where precipitation anomaly is significantly different from zero at the 95% confidence level. Numbers in brackets show the number of days in each category. The north India domain and core monsoon zone are highlighted in magenta and green, respectively.

We now investigate how precipitation and VIMF transport in the lower troposphere vary across the four clusters (Fig. 7). The two low-risk clusters are associated with reduced precipitation over most of northern India and the core monsoon zone (Fig. 7a, b). The first low-risk cluster (Fig. 7a) features anomalous easterly or northeasterly VIMF over the core monsoon zone, suggesting reduced moisture transport into the region. The anomalous VIMF over the core monsoon zone in the second low-risk cluster is westerly or northwesterly (Fig. 7b), which is associated with a weakened monsoon circulation. For the pan-India moist heat stress cluster (Fig. 7c), enhanced precipitation occurs over peninsular India, whereas there is suppressed precipitation over the eastern part of the core monsoon zone. The anomalous VIMF transport is easterly. In contrast, the northwest India moist heat stress cluster (Fig. 7d) is associated with enhanced precipitation over the core monsoon zone and features anomalous westerly VIMF over the Arabian Sea and anomalous easterlies across the IGP, resembling conditions typical of an active monsoon phase. These results are consistent with the findings of Jackson et al. (2025), who showed that in moisture-limited environments such as northwestern India, moist heatwaves tend to co-occur with enhanced precipitation. However, in energy-limited environments such as eastern India, moist heatwaves are more often associated with suppressed rainfall.

In summary, the first two EOFs of daily maximum T_w_ anomaly feature a monopole and a northwest-to-southeast dipole pattern. PC1 primarily controls the occurrence of moist heatwaves, whereas PC2 modulates their location. Moist heatwaves occur across many regions of India when PC1 > 1 and PC2 < − 1, whereas they occur only over northern India when the normalised magnitude of both PCs exceeds one. These events are typically associated with enhanced precipitation over northern India that is a moisture-limited region and suppressed precipitation over eastern India that is energy-limited.

## The relationship between moist heat and active-break events of the monsoon

We now explore moist heat’s relationship with active and break events of the monsoon. We first analyse the evolution of mean T_w_ anomalies and the occurrence of moist heatwaves during breaks and active events. We then examine how anomalies of 2 m temperature, specific humidity, and T_w_ over northern India evolve during breaks and active events. We consider mean T_w_ in this section instead of maximum T_w_, since mean T_w_ better captures the influence of minimum T_w_, which is strongly linked to moist heat stress (Di Napoli et al. 2019; Buzan and Huber 2020).

### Breaks

**Fig. 8:**
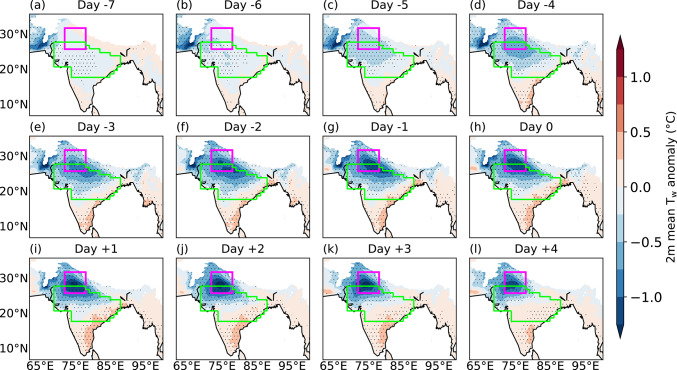
Lead-lag composites of daily mean 2 m wet-bulb temperature anomaly (T_w_; °C) for 188 breaks during June–September 1940–2023. Day 0 is the middle day of breaks. The anomalies are computed against a daily climatology during June–September 1940–2023. Stippling shows regions where T_w_ anomaly is significantly different from zero at the 95% confidence level. The north India domain and core monsoon zone are highlighted in magenta and green, respectively. Grid points over the ocean and where the mean surface pressure is less than or equal to 850 hPa are masked.

Figure 8 shows lead-lag composites of mean T_w_ anomalies for 188 breaks, with day 0 showing the middle day of breaks. Seven days prior to the middle day of breaks, weak negative T_w_ anomalies, with a magnitude of − 0.25 K, emerge over the core monsoon zone (Fig. 8a), gradually intensifying as the composite break progresses. These anomalies reach their peak strength 2–3 days after the middle day of breaks, with the strongest negative anomalies over northern India (Fig. 8j, k). In contrast, positive T_w_ anomalies of approximately 0.5 K magnitude develop over peninsular India and along the east coast, peaking about one day prior to the middle day of breaks (Fig. 8g). This confirms the findings of Ivanovich et al. (2022) that elevated T_w_ in southeastern India is linked to breaks. A persistent northwest–southeast dipole in T_w_ anomalies is evident throughout the composite period.

**Fig. 9:**
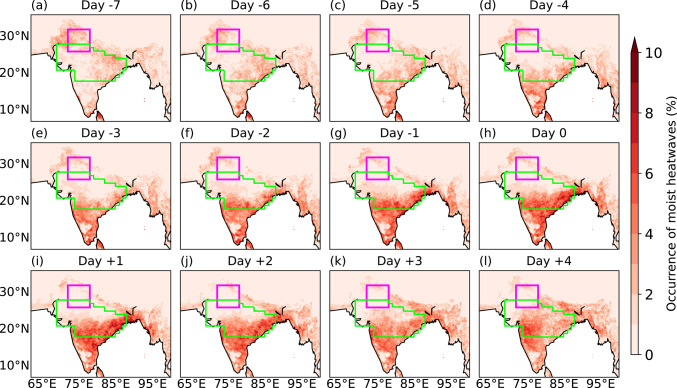
Lead-lag composites of normalised occurrence of moist heatwave days at each grid point, calculated by dividing the number of moist heatwave days by the total number of breaks (188) during June–September 1940–2023. Day 0 is the middle day of breaks. The north India domain and core monsoon zone are highlighted in magenta and green, respectively.

Figure 9 shows the corresponding lead-lag composites of moist heatwave days during breaks. Around a week prior to the middle day of breaks (Fig. 9a), moist heatwaves are sparse over the core monsoon zone, but their occurrence increases closer to the middle day of breaks. Following the middle day, the number of moist heatwave days is highest (> 10%) in peninsular India and the southern end of the core monsoon zone. In contrast, moist heatwaves remain infrequent over northwestern India, reinforcing the dipole structure seen in T_w_ anomalies.

### Active events

**Fig. 10:**
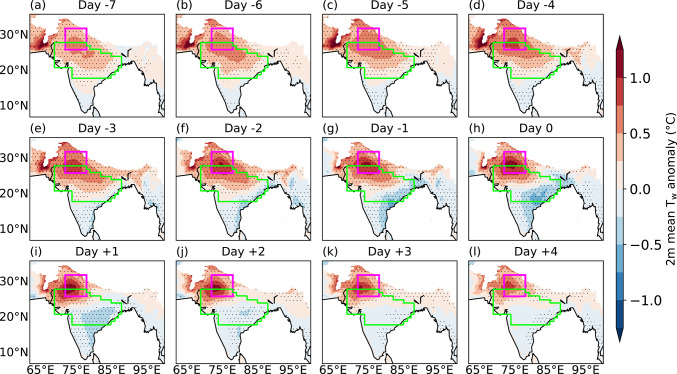
As Fig. , 8, but showing lead-lag composites for 261 active events.

Figure 10 shows lead-lag composites of mean T_w_ anomalies for 261 active events. The T_w_ anomaly pattern is reversed compared to that seen previously for breaks (Fig. 9). Around a week prior to the middle day of active events (Fig. 10a), positive anomalies of approximately 0.5 K emerge over northern India and intensify as the composite active event progresses. Around three days prior to the middle day, negative T_w_ anomalies develop along the east coast (Fig. 10e) and strengthen thereafter. At their peak, T_w_ anomalies exceed 1 K over northern India, whilst eastern India experiences negative anomalies of about − 0.5 K.

To place these T_w_ anomalies in context, we calculated the standard deviation of daily T_w_ anomalies at each grid point and each season (June–September) and averaged these values over 1940–2023. Over northern India, the mean intraseasonal standard deviation is approximately 1.5 K (not shown). The composite T_w_ anomalies associated with break and active events on their middle days reach approximately − 1.5 K and 1.5 K, respectively, indicating that these anomalies are of the order of one standard deviation. Thus, variations in T_w_ during active-break cycles represent a substantial fraction of the background intraseasonal variability and therefore constitute an important driver of moist heat stress variability.

**Fig. 11:**
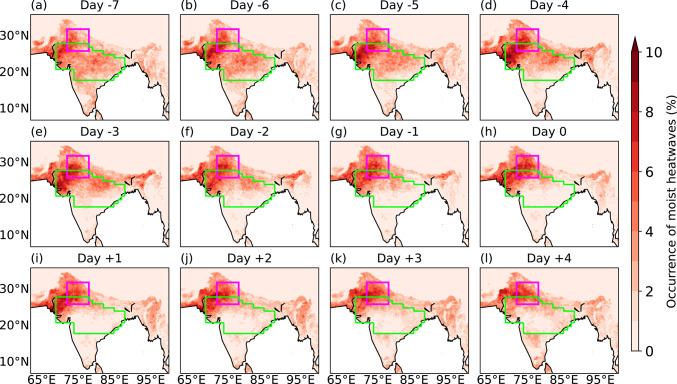
As Fig. 9, but showing lead-lag composites for 261 active events.

Throughout the composite, moist heatwave occurrence is highest over western, northwestern, and northern India, where they occur on more than 10% of days (Fig. 11). In contrast, the southern end of the core monsoon zone and peninsular India experience a lower frequency of moist heatwave days. Initially, the northern end of the core monsoon zone experiences moist heatwaves on about 6% of days, but this frequency decreases following the middle day of active events. These results suggest a spatial shift in the occurrence of moist heatwaves during active phases, indicating a pattern broadly opposite to that observed during breaks.

**Fig. 12:**
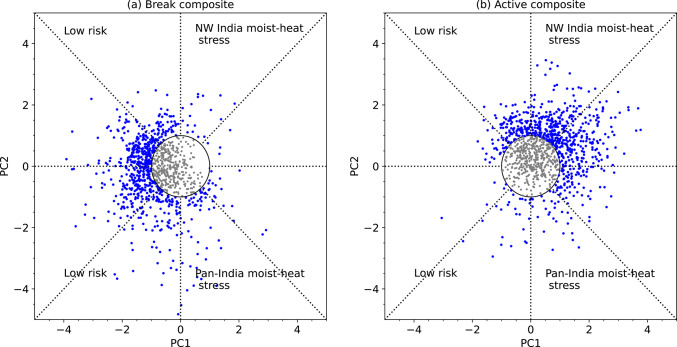
Scatter plots showing the normalised magnitude of PC1 and PC2 on all days of **a** 188 breaks and **b** 261 active events during June–September 1940–2023. Scatter points of magnitude less than or equal to one standard deviation are shown in grey.

The contrasting patterns of moist heatwave occurrence during breaks and active events can be more effectively visualised using a Wheeler–Hendon-style phase space plot (Wheeler and Hendon 2004), even though PC1 and PC2 are not dynamically coupled, unlike the pair of realtime multivariate MJO indices used therein. Figure 12a, b show a scatter plot of daily values of PC1 and PC2 during 188 break events (1045 days in total) and 261 active events (1143 days in total). The phase space is annotated using the heat stress clusters defined in Sect. 4. Whilst a few break days fall within the northwest India moist heat stress cluster, the majority are linked to a low risk of moist heat stress. In contrast, most active days are concentrated in the northwest India moist heat stress cluster, with some also occurring in the low-risk and pan-India clusters. These findings reaffirm that break periods are generally associated with a low risk of moist heat stress, whereas active phases more frequently correspond to elevated moist heat stress, especially over northwestern India.

### Evolution of thermodynamic fields over northern India

**Fig. 13:**
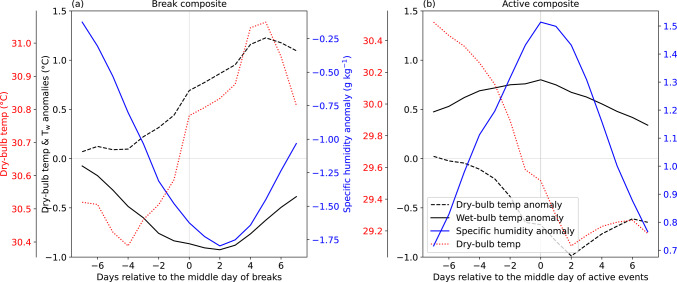
Evolution of 2 m dry bulb temperature (dotted red; °C) and anomalies of 2 m dry bulb temperature (dashed black; °C), 2 m wet-bulb temperature (T_w_ with unit °C; solid black) and 2 m specific humidity (solid blue; g kg.^−1^) for a composite of (left) 188 breaks and (right) 261 active events. The results are shown for the north India domain (26°–32°N, 72°–78°E). Day 0 shows the middle day of breaks and active events. All anomalies are computed against a daily climatology computed over June–September 1940–2023.

We now turn our attention to examining the evolution of 2 m dry-bulb temperature and specific humidity anomalies to assess their relative influence on T_w_, focusing on the north India domain where T_w_ anomalies are most pronounced. During breaks, the absolute and anomalous dry-bulb temperature increase as the break progresses (Fig. 13a), which is likely due to reduced cloud cover and enhanced insolation. Specific humidity, however, decreases as the middle day of breaks approaches. T_w_ anomalies become increasingly negative, and strongest negative anomalies occur two days after the middle day of breaks, mirroring the decline in specific humidity. In contrast, dry-bulb temperature decreases due to reduced insolation, and its anomaly becomes negative during the active phase (Fig. 13b). The lowest dry-bulb temperature and its largest negative anomaly are observed two days following the middle day of the composite active event. However, the specific humidity anomaly increases and peaks around the middle day of the composite active event, and the T_w_ anomaly follows a similar pattern. This suggests that specific humidity has a stronger influence on T_w_ variability than dry-bulb temperature, consistent with previous findings (e.g., Sherwood 2018; Ivanovich et al. 2022, 2024; May et al. 2022, 2025).

In summary, moist heatwaves occur more frequently over peninsular and eastern India during monsoon breaks, coinciding with negative T_w_ anomalies over northern India. This forms a northwest-to-southeast dipole pattern. In contrast, active events are associated with strong positive T_w_ anomalies and increased moist heatwave frequency over northern and northwestern India, alongside reduced T_w_ and moist heatwave activity in peninsular India, effectively reversing the break-phase pattern. Break days are largely characterised by low moist heat stress, whereas active days are predominantly linked to widespread occurrence of moist heatwaves in northern and northwestern India. Further analysis indicates that specific humidity plays a more influential role than dry-bulb temperature in shaping T_w_ anomalies over northern India during the active and break events of the monsoon.

## The role of the BSISO in modulating the occurrence of moist heatwaves

**Fig. 14:**
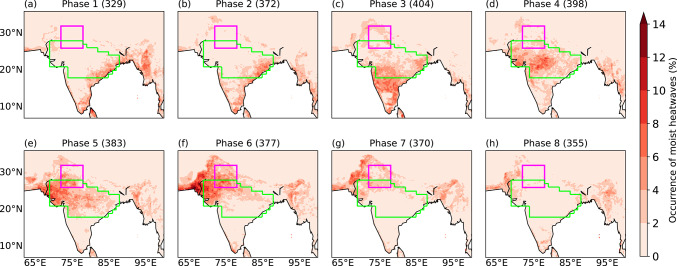
The frequency of occurrence of moist heatwave days at each grid point (% days) during the eight phases of the Boreal Summer Intraseasonal Oscillation (BSISO). The frequency at each grid point in a BSISO phase is normalised by the number of days of occurrence of the BSISO in that phase (shown in brackets in the title). Only those days are considered during which the BSISO amplitude exceeded one standard deviation over the period June–September 1979–2021.

Since the BSISO modulates active-break phases of monsoon rainfall, and active-break phases modulate the occurrence of moist heatwaves, we expect some relationship between the BSISO and the occurrence of moist heatwaves. Figure 14 shows the frequency of occurrence of moist heatwave days in the eight BSISO phases. During phases 1 and 2 (Fig. 14a, b), the occurrence of moist heatwaves is primarily confined to peninsular India and the east coast, where the frequency of moist heatwave days is about 8%. Over peninsular India, the frequency of moist heatwave days is largest in phase 3 (Fig. 14c). As the BSISO propagates northwards, the region of occurrence of moist heatwaves shifts towards the core monsoon zone, particularly in phases 4 and 5 (Fig. 14d, e). Their occurrence is largest over northwestern India and adjoining eastern Pakistan in phases 5 and 6 (Fig. 14e, f). In these phases, there is a strong decrease in the frequency of moist heatwave days over eastern and peninsular India. Moist heatwaves continue to occur over northern and northwestern India in phase 7 (Fig. 14g). Additional analysis of mean dry-bulb temperature and specific humidity anomalies at 2 m (not shown) reveals that during BSISO phases 5–7, specific humidity anomaly over northern and northwestern India exceeds 2 g kg^−1^, even as dry-bulb temperature anomaly remains relatively weak in magnitude. This underscores the dominant influence of increased humidity, which is linked to the convectively active phase of the BSISO, in modulating T_w_, consistent with the findings of Ivanovich et al. (2022).

**Fig. 15:**
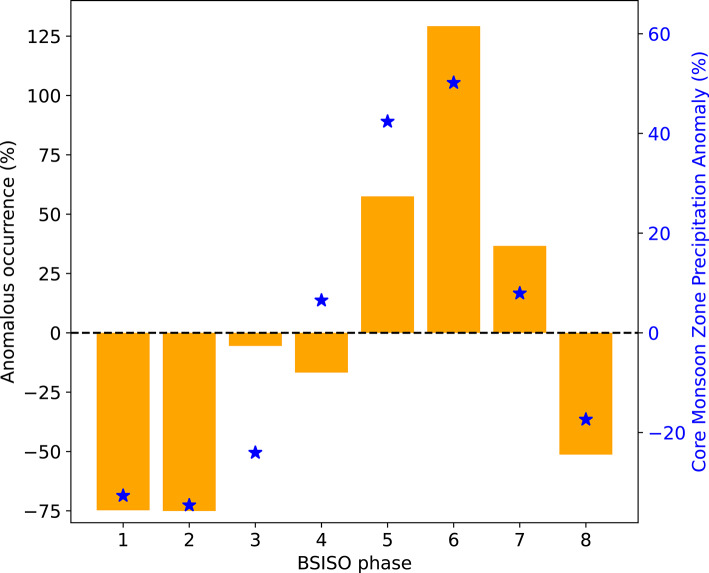
Anomalous number of grid points (%) in the north India domain (26°–32°N, 72°–78°E) featuring moist heatwaves during phases of the Boreal Summer Intraseasonal Oscillation (BSISO). The total number of grid points in the domain is 625. Counts in each phase are normalised by the occurrence of the BSISO in that phase. A 0% value indicates that the frequency of grid points with moist heat waves equals the climatological expected value, whereas 100% indicates twice that of the climatological mean value. A–100% value would indicate the absence of any grid points with moist heat waves. Blue markers show the precipitation anomaly (%) over the core monsoon zone in each BSISO phase. Moist heatwaves and precipitation are retained only if they occurred when the BSISO amplitude exceeded one standard deviation during June–September 1979–2021.

To better understand this result, we now focus on the north India domain, which has a total of 625 grid points in ERA5. We first compute the mean number of grid points experiencing moist heatwaves across all eight BSISO phases. For each phase, we then calculate the anomalous moist heatwave occurrence by subtracting this mean from the number of grid points featuring moist heatwaves in that phase, and normalise the difference by the mean. The occurrence of moist heatwaves is suppressed in BSISO phases 1–4, as indicated by the negative anomalies. It peaks in BSISO phase 6 during which the anomalous occurrence exceeds 125% (Fig. 15). This peak coincides with the occurrence of the peak active phase of the monsoon, which is indicated by the largest positive precipitation anomaly. The anomalous occurrence of moist heatwaves over northern India then gradually declines in subsequent BSISO phases.

## Conclusions

Moist heat increases health risks by disrupting the human body’s ability to regulate temperature through sweating, leading to greater physiological heat stress. Despite its growing relevance, there has been a limited meteorological analysis of moist heat globally, including in India, where the monsoon delivers substantial moisture between June and September. This study aims to address this gap by analysing the characteristics and drivers of moist heat in India using the ERA5 reanalysis for the period June–September 1940–2023. The primary metric used is the 2 m wet-bulb temperature (T_w_). The key findings are summarised below.

### Spatio-temporal patterns of variability of moist heat

We performed an EOF analysis of daily maximum T_w_ anomalies. The leading two EOF modes feature a monopole and northwest-to-southeast dipole structure, respectively. They are similar to the first two EOF modes identified previously in dry-bulb temperature studies (e.g.Ratnam et al. 2016; Lekshmi and Chattopadhyay 2022). We found that the first principal component (PC1) controls the frequency of occurrence of moist heatwaves, whereas PC2 controls their location. The maximum T_w_ across most of India is elevated when PC1 > 1 and PC2 < − 1, with the highest maximum T_w_ occurring over the Indo-Gangetic Plains and northern India. This leads to a widespread occurrence of moist heatwaves across the country, including the core monsoon zone. In contrast, the occurrence of moist heatwaves remains confined to northern India and adjoining eastern Pakistan when both PC1 > 1 and PC2 > 1. Moist heatwaves do not occur in India when PC1 < − 1.

### The relationship between active-break phases of the monsoon and moist heat

**Fig. 16:**
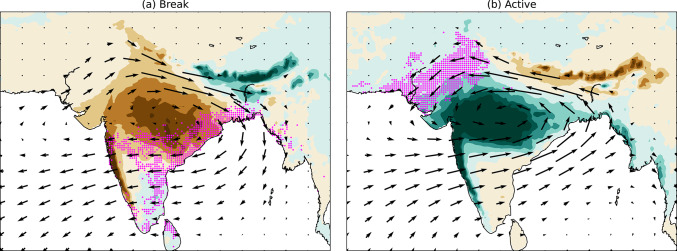
Schematic diagram illustrating the preferred location of moist heatwaves (magenta dots) on the middle day of **a** breaks and **b** active events during June–September 1940–2023. Only those locations are shown where the relative occurrence of moist heatwave days exceeds 5% of 188 and 261 middle days of breaks and active events, respectively. Shading shows precipitation anomaly, with brown showing negative precipitation anomaly and green showing positive precipitation anomaly. Vectors show the 1000–700 hPa vertically integrated moisture flux during the same period.

We found that active and break phases of monsoon rainfall modulate the occurrence of moist heat and moist heatwaves in India. During breaks, moist heatwaves are more frequent over peninsular and eastern India, whereas during active phases, they are more frequent over northern and northwestern India. This is illustrated schematically in Fig. 16. Given that northwestern India is typically moisture-limited and eastern India energy-limited, these patterns reinforce the distinction between moisture and energy-limited environments for moist heatwaves, as discussed by Jackson et al. (2025). Additionally, we found that specific humidity exerts a stronger influence on T_w_ than dry-bulb temperature, in agreement with previous studies (e.g., Ivanovich et al. 2022, 2024; May et al. 2022, 2025).

### Modulation of moist heatwaves by the BSISO

We examined how the BSISO modulates the occurrence of moist heatwaves in the country. We found that when the convectively enhanced phase of the BSISO is over Sri Lanka and peninsular India (i.e., BSISO phases 1–4), the occurrence of moist heatwaves over northern India is suppressed, and moist heatwaves are confined to peninsular India and parts of the core monsoon zone (i.e., central India). Over subsequent phases, their occurrence over northern and northwestern India is enhanced when the convectively active phase of the BSISO shifts northwards. The highest occurrence of moist heatwave days over northern India is observed during BSISO phase 6, which coincides with the largest positive precipitation anomalies over the core monsoon zone and often corresponds to an active monsoon phase. These findings are consistent with Ivanovich et al. (2022), who also identified modulation of moist heat by tropical intraseasonal oscillations.

The results of this study reveal important characteristics of moist heatwaves in India. The modulation of moist heatwave occurrence by the first two PCs and the BSISO presents promising opportunities for developing early warning and forecasting tools. Such advancements could greatly assist stakeholders, such as government agencies, in improving preparedness and mitigating health risks during periods of heightened moist heat stress in India.

A limitation of this study is that we did not analyse teleconnection patterns that could cause moist heatwaves in India. In a future study, researchers could explore this aspect and consider frameworks used previously for the analysis of dry heat (e.g., Ratnam et al. 2016; Rohini et al. 2016; Lekshmi and Chattopadhyay 2022). Another limitation is that we did not incorporate alternative datasets such as quality-controlled station observations or other reanalysis products to calculate T_w_. In a future study, researchers could consider quality-controlled station data (e.g., the Met Office Hadley Centre Integrated Surface Database; Dunn et al. 2012) or the high-resolution IMDAA reanalysis (Rani et al. 2021). Moreover, they could also examine the predictability of moist heatwaves on the subseasonal-to-seasonal time scales, since that would immensely benefit stakeholders in India. For such a study, they could consider hindcasts from the Met Office (e.g., GloSea; MacLachlan et al. 2015) or the National Centre for Medium-Range Weather Forecasting (NCMRWF) Unified model (NCUM).

## Electronic supplementary material

Below is the link to the electronic supplementary material.

Supplementary file 1 (PDF 2145 kb). Supplememtary Figures S1–S8. They show scatter plots of monthly means of dry-bulb temperature, specific humidity, and wet-bulb temperature; the 95th percentile of daily maximum wet-bulb temperature for different months of the monsoon; lead-lag correlation between the first two principal components (PCs); maximum Heat Index in various combinations of PCs; a box-and-whisker plot showing the maximum wet-bulb temperature over northern India for the various combinations of PCs; mean wet-bulb temperature in various combinations of PCs; and anomalies of daily mean dry-bulb temperature and specific humidity in various combinations of PCs.

## Data Availability

The ERA5 hourly data on pressure levels is available at 10.24381/cds.bd0915c6. The Python code to calculate the Davies-Jones wet-bulb temperature is available at https://github.com/cr2630git/wetbulb_dj08_spedup. The Thermofeel Python library (Brimicombe et al. 2022; https://thermofeel.readthedocs.io/ en/latest/) was used to calculate the adjusted Heat Index. The high-resolution IMD gridded dataset is available at https://imdpune.gov.in/cmpg/Griddata/Rainfall_25_ NetCDF.html. The catalogue of moist heatwaves is available upon request from Akshay Deoras.

## Footnotes

1 Jackson et al. (2025) used daily mean T_w_ for identifying moist heatwaves, but they showed that their results were insensitive to whether daily maximum or daily mean T_w_ was used.

2 Breaks occurring during June and September are generally ignored to prevent contamination of the results by progressions of the onset and withdrawal of the monsoon. However, we do not eliminate such breaks in our study, since our objective is to analyse the climatology of breaks and their role in triggering moist heat waves irrespective of the month of occurrence. Thus, the exclusion of breaks occurring during June and September is not necessary for this study.
