# Supplementary material for: Anatomy of moist heatwaves in India during the summer monsoon season
Source: Clim Dyn. 2026 Feb 16;64(3):103. doi: 10.1007/s00382-025-08023-w (PMC12909335; doi:10.1007/s00382-025-08023-w)
Supplement: Supplementary file 1 — (PDF 2145 kb). Supplememtary Figures S1–S8. They show scatter plots of monthly means of dry-bulb temperature, specific humidity, and wet-bulb temperature; the 95th percentile of daily maximum wet-bulb temperature for different months of the monsoon; lead-lag correlation between the first two principal components (PCs); maximum Heat Index in various combinations of PCs; a box-and-whisker plot showing the maximum wet-bulb temperature over northern India for the various combinations of PCs; mean wet-bulb temperature in various combinations of PCs; and anomalies of daily mean dry-bulb temperature and specific humidity in various combinations of PCs. [file 382_2025_8023_MOESM1_ESM.pdf]

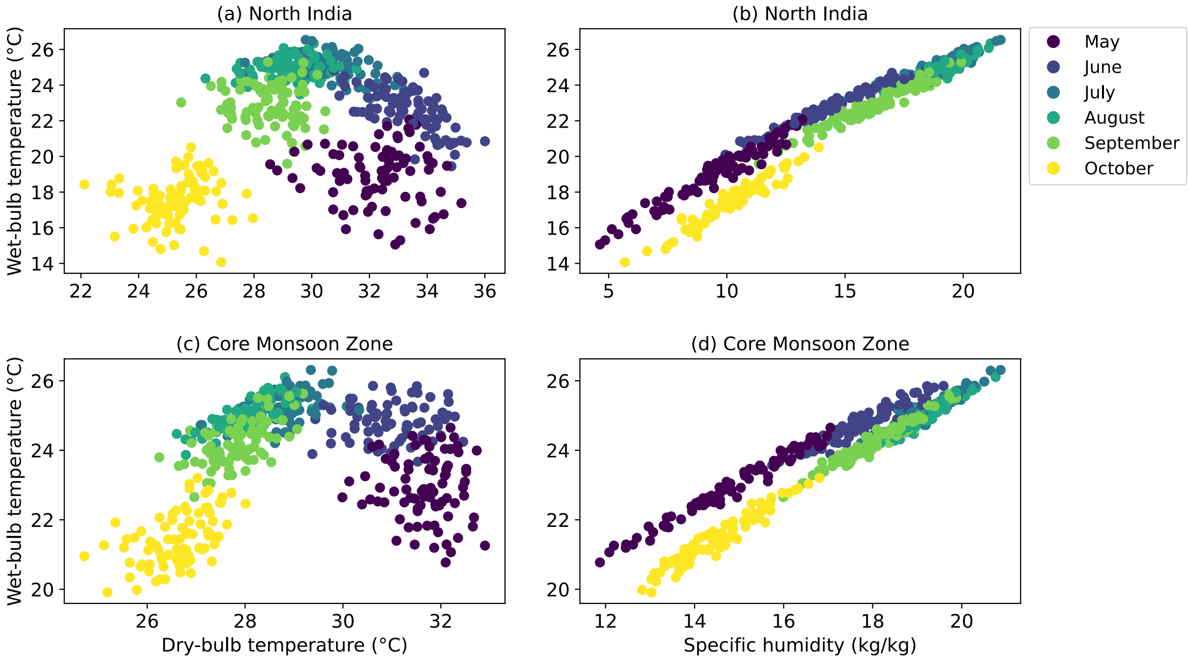


Figure S1. Scatter plots showing the relationship between monthly means of daily mean 2 m dry-bulb temperature and wet-bulb temperature (a and c) and 2 m specific humidity and wet-bulb temperature (b and d) for the North India domain (a and b; 26°N–32°N, 72°E–78°E) and core monsoon zone (c and d). The period between 1940 and 2023 is considered for this analysis. Scatter points are coloured as per the month.

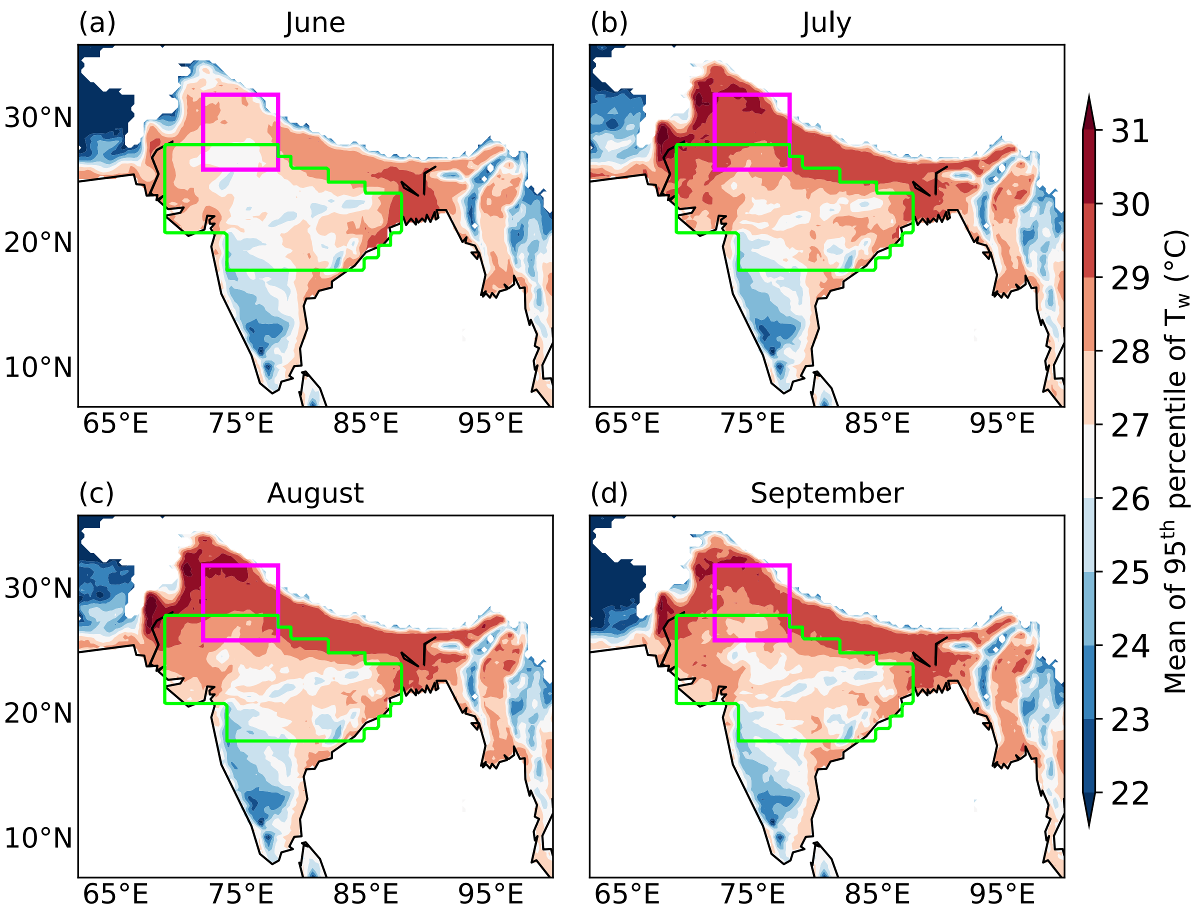


Figure S2. Mean of 95th percentile of daily maximum 2 m wet-bulb temperature ($T_w$; °C) during 1940–2023. The north India domain and core monsoon zone are highlighted in magenta and green, respectively.

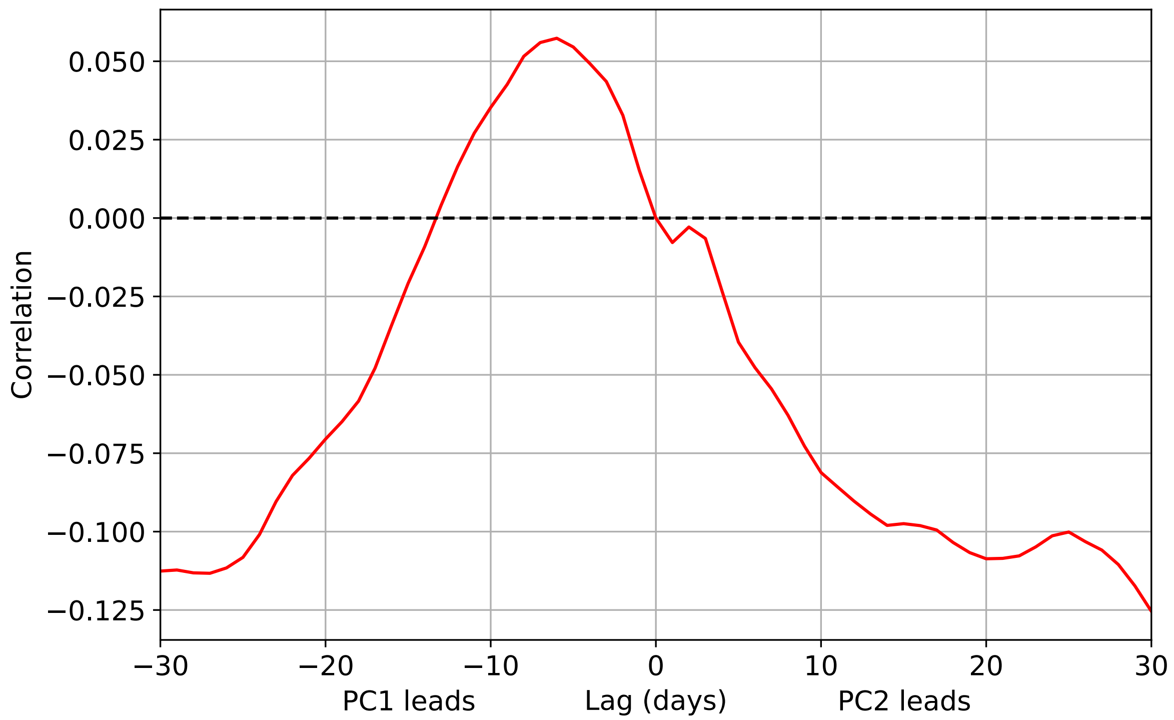


Figure S3. Lead-lag correlation between PC1 and PC2 of daily maximum 2 m wet-bulb temperature anomaly during June–September 1940–2022.

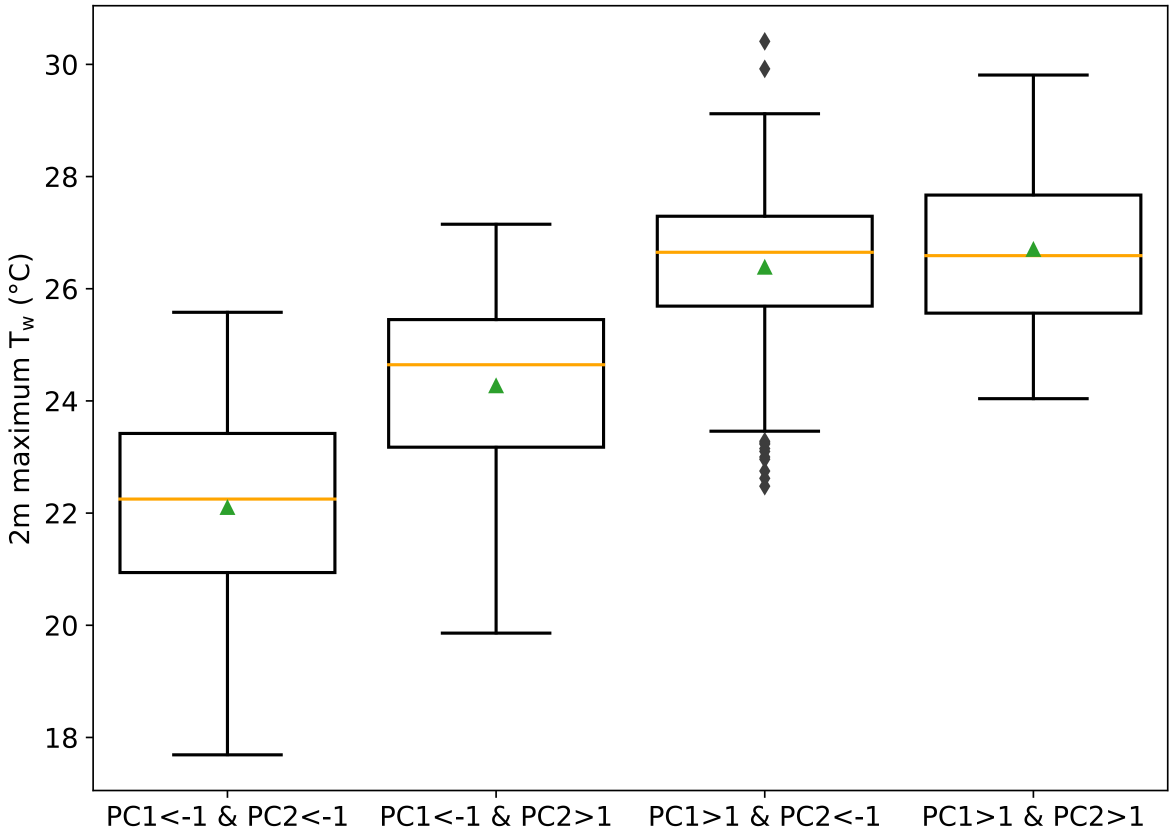


Figure S4. Box and whisker plot showing the domain mean of maximum 2 m wet-bulb temperature ($T_w$; °C) identified at each grid point in the north India domain (26°–32°N, 72°–78°E) for various combinations of principal components (PCs) during June–September 1940–2023. Green triangles, orange lines, and black diamonds show the domain mean of maximum $T_w$, median of maximum $T_w$, and outliers, respectively.

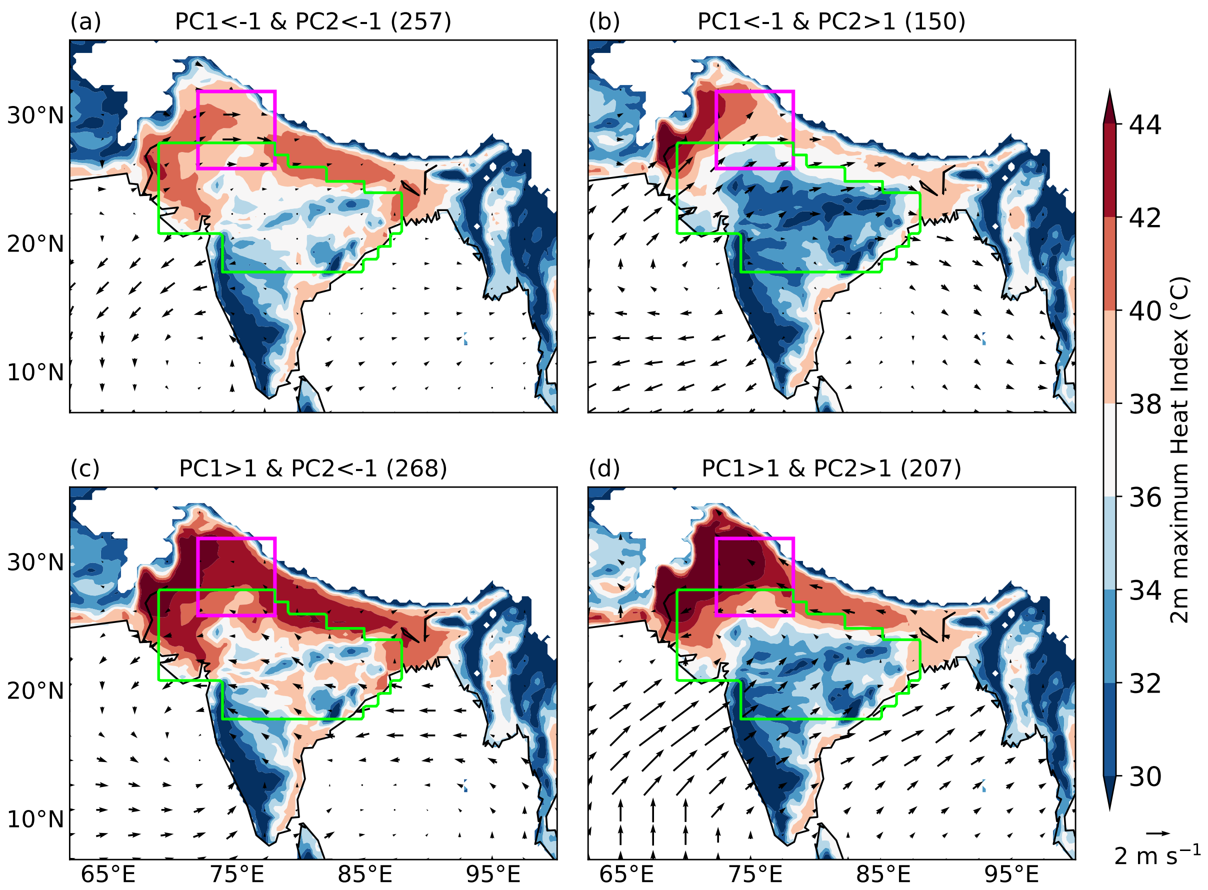


Figure S5. As Figure 5, but showing 2 m maximum Heat Index (shading; °C). Note the different contour interval.

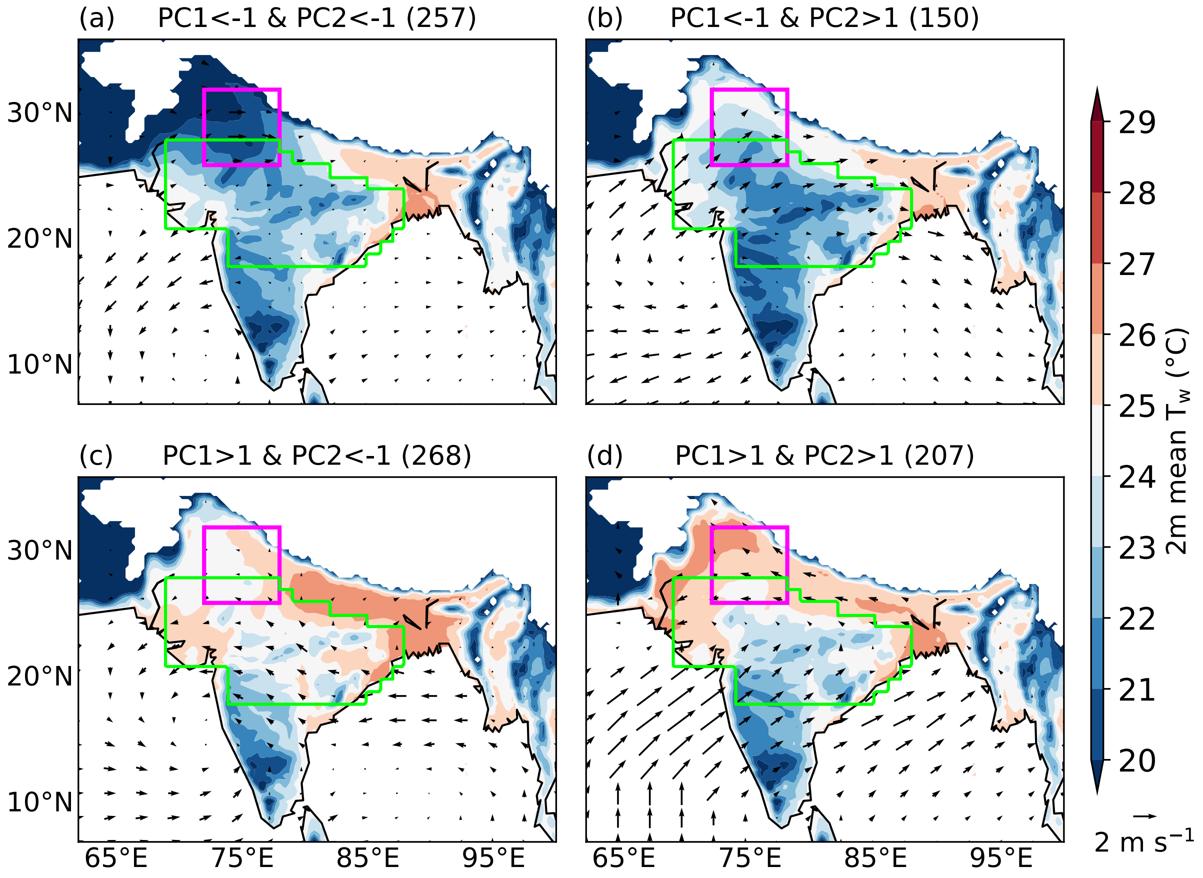


Figure S6. As Figure 4, but showing mean $T_w$.

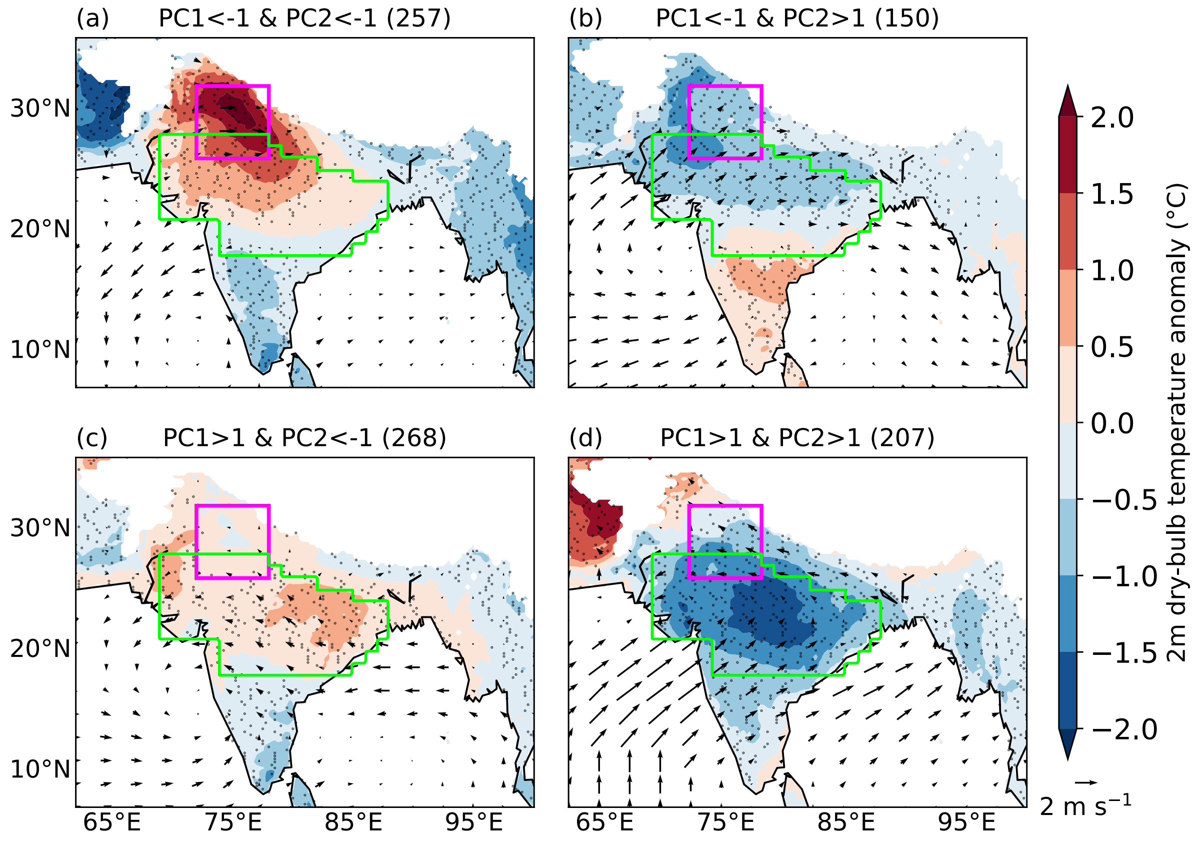


Figure S7. As Figure 4, but showing anomalies of daily mean 2 m dry-bulb temperature (shading; °C). Stippling shows regions where temperature anomaly is significantly different from zero at the 95% confidence level.

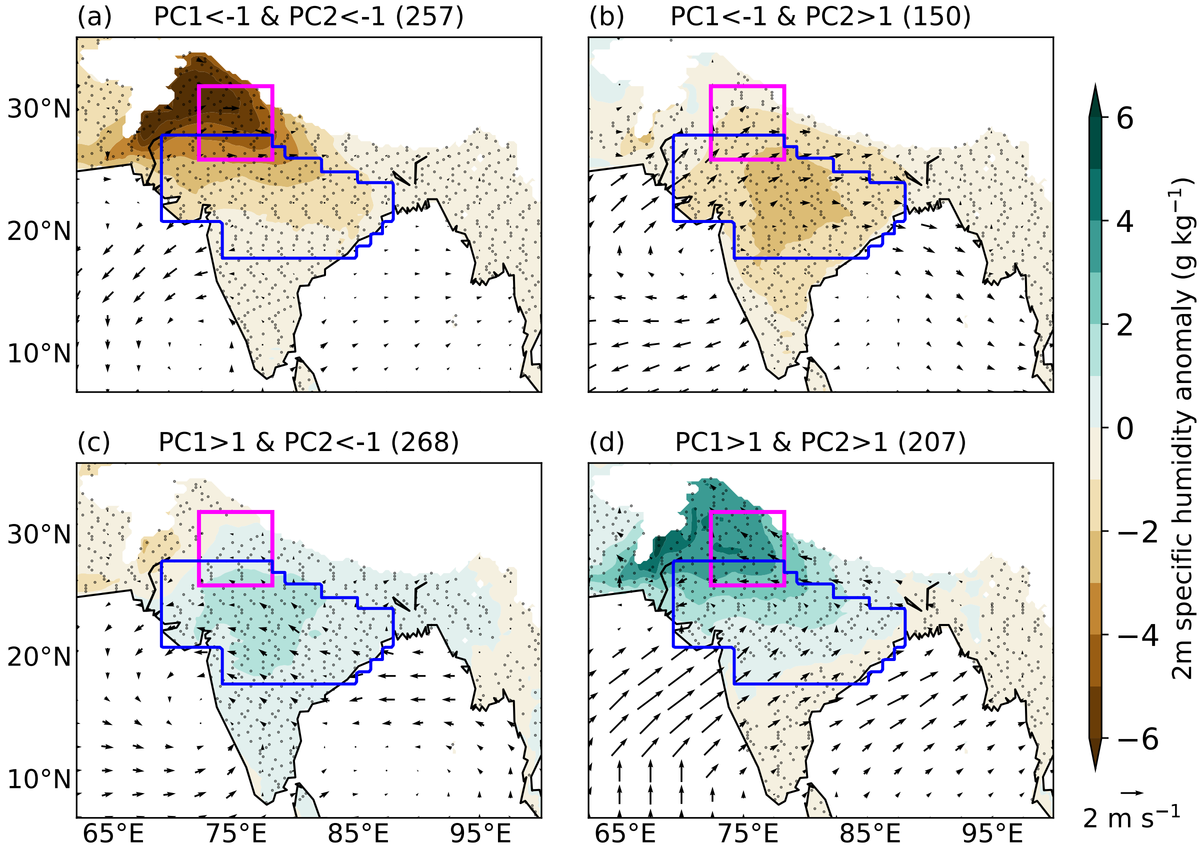


Figure S8. As Figure 4, but showing anomalies of daily mean 2 m specific humidity (shading; g kg$^{-1}$). Note that the north India domain and core monsoon zone are highlighted in magenta and blue, respectively. Stippling shows regions where specific humidity anomaly is significantly different from zero at the 95% confidence level.
